# Health-related quality of life among adolescents with allergy-like conditions – with emphasis on food hypersensitivity

**DOI:** 10.1186/1477-7525-2-65

**Published:** 2004-11-19

**Authors:** Birgitta Marklund, Staffan Ahlstedt, Gun Nordström

**Affiliations:** 1 Centre for Allergy Research, Karolinska Institutet, S-171 77 Solna, Sweden; 2 Department of Nursing, 23300, Karolinska Institutet, S-141 83 Huddinge, Sweden; 3 National Institute of Environmental Medicine, Karolinska Institutet, S-171 77 Solna, Sweden; 4 Division of Health and Caring Sciences, Karlstad Universitet, S-651 88 Karlstad, Sweden

**Keywords:** Health-related quality of life, hypersensitivity, allergic disease, food hypersensitivity, adolescence, gender

## Abstract

**Background:**

It is known that there is an increase in the prevalence of allergy and that allergic diseases have a negative impact on individuals' health-related quality of life (HRQL). However, research in this field is mainly focused on individuals with verified allergy, i.e. leaving out those with self-reported allergy-like conditions but with no doctor-diagnosis. Furthermore, studies on food hypersensitivity and quality of life are scarce. In order to receive information about the extent to which adolescent females and males experience allergy-like conditions and the impact of these conditions on their everyday life, the present study aimed to investigate the magnitude of self-reported allergy-like conditions in adolescence and to evaluate their HRQL. Special focus was put on food hypersensitivity as a specific allergy-like condition and on gender differences.

**Methods:**

In connection with lessons completed at the children's school, a study-specific questionnaire and the generic instrument SF-36 were distributed to 1488 adolescents, 13–21 years old (response rate 97%).

**Results:**

Sixty-four per cent of the respondents reported some kind of allergy-like condition: 46% reported hypersensitivity to defined substances and 51% reported allergic diseases (i.e. asthma/wheezing, eczema/rash, rhino-conjunctivitis). A total of 19% reported food hypersensitivity. Females more often reported allergy-like conditions compared with males (p < 0.001). The adolescents with allergy-like conditions reported significantly lower HRQL (p < 0.001) in seven of the eight SF-36 health scales compared with adolescents without such conditions, regardless of whether the condition had been doctor-diagnosed or not. Most adolescents suffered from complex allergy-like conditions.

**Conclusions:**

The results indicate a need to consider the psychosocial impact of allergy-like conditions during school age. Further research is needed to elucidate the gender differences in this area. A team approach addressing better understanding of how allergy-like conditions impair the HRQL may improve the management of the adolescent's health problems, both in health-care services and in schools.

## Background

An increase in the prevalence of asthma and atopy during the last two decades is documented for both children [1] and adults [2]. The International Study of Asthma and Allergies in Childhood (ISAAC) has demonstrated a large variation in the prevalence of asthma symptoms in children throughout the world [3]. An ISAAC-study on prevalence of childhood allergic diseases in Scandinavia and Eastern Europe has shown that the prevalence among Swedish children 13–14 years of age is 15% for asthma/wheezing, 17% for eczema and 26% for rhino-conjunctivitis [4].

It is well known that there are more individuals with perceived hypersensitivity than individuals with verified/doctor-diagnosed allergy. This is especially true when it comes to perceived food hypersensitivity with up to tenfold higher figures versus verified food allergy [5,6]. Prevalence figures for food hypersensitivity vary considerably (1–25%) with regard to study design, population of subjects [7-9] and country [7,8].

Research has demonstrated that allergic diseases have a negative impact on individuals' health-related quality of life (HRQL) and a number of studies describe HRQL-deteriorations in children and adults with asthma [10-12], eczema [13-15] and rhinitis [16-18]. Studies have also shown that allergy-associated physical and organ-related measures and tests do not always correlate with HRQL-scores [11,12].

Adverse reactions to food constitute an important part of allergy-associated problems, especially among children. Still, studies on food allergy and HRQL are scarce. However, parental perception of physical and psychosocial functioning, measured with the Children's Health Questionnaire (CHQ-PF50), has shown that childhood food allergy has a significant emotional impact on the parent and limits the family activities [19]. It has also been documented that, from a parental perspective, children with peanut allergy have significantly more disruption in their daily life compared with children with rheumatological disease, due to their children's risk of death [20]. Furthermore, peanut allergic children have been shown to report more anxiety about eating and more fear of an adverse event compared with children with diabetes mellitus [21].

Previous studies have shown that adolescent males have a higher prevalence of atopy than adolescent females [22] although there is a female dominance in self-reported allergic diseases [23]. Furthermore, the prevalence of asthma has been found to vary with age and sex, showing a male predominance before puberty that changes into a higher prevalence in females in adolescence [24,25].

Research in the allergy field is mainly focused on individuals with verified allergy and their suffering from these conditions, i.e. leaving out those with self-reported allergy-like conditions but with no doctor-diagnosis. Still, one can presume that perceived allergy could have an impact on the health-related quality of life (HRQL) and involve suffering, regardless of verifiable diagnosis.

In order to obtain information about the extent to which adolescent females and males themselves experience allergy-like conditions and the impact of these conditions on their everyday life, the present study aimed to investigate the magnitude of self-reported allergy-like conditions in adolescence and to evaluate their HRQL. Special focus was put on food hypersensitivity as a specific allergy-like condition and on gender differences.

## Methods

### Subjects and procedure

The present study involved adolescents at the senior level of the nine-year compulsory school and at the upper secondary school in a municipality in the south of Stockholm, Sweden. The socio-demographic distribution of the inhabitants in this municipality was slightly above in socio-economics and slightly below in number of immigrants compared with the country as a whole. A total of 2064 adolescents were registered in the schools at the relevant levels, all with the Swedish language as their school language.

One week prior to starting the study (May 2003), an information letter outlining the purpose of the investigation, including an assurance of confidentiality and voluntary participation, was distributed to both the adolescents (n = 2064) and their parents. In connection with lessons at school, the teachers distributed questionnaires to the 1488 adolescents who were present at school. The instructions given by the teachers and the administration of the questionnaires were standardized. Two questionnaires were distributed together in one envelope, with the HRQL-questionnaire (se below) at the top. The adolescents themselves completed the questionnaires during that particular lesson. No other support was offered than the possibility to ask the teacher clarifying questions regarding the wording.

After completion of the questionnaires, or in case of renouncing participation, each adolescent put the questionnaires into the envelope, sealed it, and handed it over to the teacher, who forwarded the envelopes to one of the authors (BM). The discrepancy between the number of registered adolescents (n = 2064) and the number, who were actually present when the data was collected (n = 1488), was partly due to the fact that it was close to the summer holiday and graduation and some adolescents attended activities outside school. The school records confirmed their absences. As 37 adolescents had not properly filled in the questionnaires, those were excluded from the study. In total 1451 questionnaires (97%) remained for data analysis. Age ranged between 13 and 21 years (mean 16.2 years), with 99% of the adolescents between 14 and 20 years. In total 696 females and 716 males had reported their gender. For 39 adolescents the gender was not reported.

The terminology used in this study is according to ISAAC (The International Study of Asthma and Allergies in Childhood) [3] and EAACI (The European Academy of Allergology and Clinical Immunology) position paper [26] (Table 1).

**Table 1:** Terminology and definitions^1^

**Allergy-like conditions**			
**Hypersensitivity**	**Allergic diseases**		
	Asthma/wheezing	Eczema/rash	Rhino-conjunctivitis
	
Self-reported hypersensitivity, allergy or intolerance to food or other defined environmental substances.	Self-reported asthma, wheezing or whistling in the chest.	Self-reported recurrent eczema or itchy rash for at least six months.	Self-reported sneezing, runny nose, blocked nose or itchy-watery eyes without a cold.

^1^Terminology according to ISAAC [3] and EAACI position paper [26].

### Questionnaires

A study-specific questionnaire was used to evaluate the magnitude of allergy-like conditions during the past twelve months and to evaluate the frequency of allergy testing. The questionnaire, devised by the authors, was based on relevant literature on similar subjects [3]. Prior to the data collection, a pilot test of the questionnaire was performed with fourteen adolescents, who were not included in the present study, and subsequently minor lexical adjustments were made. Some of the questions included were as follows:

"Are you allergic or hypersensitive to any of the following?" (Possible answers: furred animal, pollen, dust/mite, food, nickel, other substances: Yes/No.)

"If you are allergic or hypersensitive to any food items, what reactions or symptoms do you perceive? (Possible answers: not allergic or hypersensitive, eczema, rash, eye-nose-symptoms, itchy mouth, breathing difficulties, vomiting-diarrhoea-stomach ache, allergic chock, other)."

"Have you had asthma, wheezing or whistling in the chest in the past 12 months? (Yes/No)"

"In the past 12 months, have you had recurrent eczema or itchy rash for at least 6 months? (Yes/No)"

"In the past 12 months, have you had a problem with sneezing, or a runny, or a blocked nose when you did not have a cold? (Yes/No)"

"In the past 12 months, have you had a problem with itchy-watery eyes when you did not have a cold? (Yes/No)"

The questions concerning allergic diseases were taken verbatim from the ISAAC study [3].

The generic instrument Medical Outcome Trust Short Form 36 Health Survey (SF-36) was used to measure HRQL. SF-36 is a well-validated and reliable measure of HRQL in adults and adolescents from the age of 14, and normative data are available for the Swedish population [27]. The SF-36 consists of 36 items, which refer to eight health scales related to daily life activities. Four of these health scales represent the physical dimension and the remaining four health scales represent the mental dimension of the HRQL concept. The scale scores range from 0 to 100, with 100 representing the highest level of functioning and well being [27,28]. The footnote of Table 4 conveys a summary of the contents in the SF-36 health scales.

**Table 4:** Comparison of SF-36 scores between adolescents with and without allergy-like conditions and between females and males with allergy-like conditions

	Allergy-like conditions		No such conditions			Females with allergy-like conditions		Males with allergy-like conditions		
	N = 931		N = 520			N = 501		N = 412		

SF-36 health scales^1^	Mean	SD	Mean	SD	*p*-value	Mean	SD	Mean	SD	*p*-value

**Physical dimension**										
Physical functioning (PF)	91.1	16.4	91.7	19.1	NS	91.1	13.8	91.2	18.8	NS
Role functioning-physical (RP)	75.7	31.3	83.2	27.2	<0.001	73.6	31.5	78.4	30.4	<0.05
Bodily pain (BP)	71.3	23.6	81.2	20.3	<0.001	67.9	24.1	75.3	22.4	<0.001
General health (GH)	69.6	20.5	80.8	16.7	<0.001	66.3	21.1	73.5	19.2	<0.001
**Mental dimension**										
Vitality (VT)	53.0	21.0	62.2	21.0	<0.001	50.2	20.5	56.4	21.2	<0.001
Social functioning (SF)	81.3	21.6	86.9	18.9	<0.001	79.1	22.0	84.1	20.8	<0.001
Role functioning-emotional (RE)	65.4	40.3	75.4	36.8	<0.001	58.2	42.1	73.7	36.3	<0.001
Mental health (MH)	67.3	19.6	74.9	19.1	<0.001	62.7	20.2	72.8	17.4	<0.001

^1^**Summary of contents of SF-36 health scales: ***Physical dimension*: PF = Ability to perform daily physical activities, e. g. walking, running, lifting, and other moderate physical efforts; RP = Extent to which physical health limits work/daily activities; BP = Intensity of pain and its interference with normal activities; GH = Personal evaluation of general health status, presently and in the future.

*Mental dimension*: VT = Personal evaluation of energy, tiredness, etc; SF = Extent to which physical health or emotional problems interfere with normal social activities; RE = Extent to which emotional problems limit work/daily activities; MH = Personal evaluation of mental health, including anxiety, depression, and general positive and negative affects [27, 28].

### Statistical analyses

For statistical analyses the SPSS 11.0 program was used. SF-36 data was processed by means of an SPSS program provided by the HRQL-group at the University of Gothenburg, Sweden [27]. Internal consistency of the SF-36 health scales was tested by means of Cronbach's alpha and in this study ranged between 0.72 and 0.91, with the exception of the scale for social functioning (SF) showing 0.61.

To test differences in proportions between groups, the Chi-square test was used. The Student's t-test and, when appropriate, the one-way analysis of variance (ANOVA) were used to assess differences in means between groups. A p-value <0.05 was considered to be statistically significant.

### Ethical approval

This study was approved by the Director of School Administration in Tyresö municipality and by the research ethics committee at Huddinge University Hospital.

## Results

### Allergy-like conditions

As shown in Table 2, a total of 931 adolescents (64%) reported that they suffered from some kind of allergy-like condition, i.e. either hypersensitivity to defined substances (46%) or allergic diseases (51%) (definitions given in Table 1). In particular, 19% of the whole group (24% of the females and 14% of the males) reported that they reacted to some food (Table 2).

**Table 2:** Self-reported allergy-like conditions among the 1451 adolescents

	Total		Females		Males		
	
	N = 1451^1^		N = 696		N = 716		*p*-values
	
	N	(%)	N	(%)	N	(%)	
	
**Allergy-like conditions, totals**	**931**	**(64)**	**501**	**(72)**	**412**	**(58)**	**<0.001**
*Hypersensitivity to defined substances*	*663*	*(46)*	*364*	*(52)*	*282*	*(39)*	<*0.001*
pollen	335	(23)	161	(23)	162	(23)	NS
**food**	**271**	**(19)**	**165**	**(24)**	**98**	**(14)**	**<0.001**
furred animal	234	(16)	117	(17)	110	(15)	NS
dust/mite	194	(13)	114	(16)	75	(11)	<0.001
nickel	169	(12)	132	(19)	32	(5)	<0.001
other substances^2^	94	(7)	62	(9)	31	(4)	<0.001
*Allergic diseases*	*739*	*(51)*	*408*	*(59)*	*319*	*(45)*	<*0.001*
asthma/wheezing	231	(16)	156	(22)	72	(10)	<0.001
eczema/rash	286	(20)	178	(26)	105	(15)	<0.001
rhino-conjunctivitis	546	(38)	287	(41)	248	(35)	<0.05

^1 ^Gender unknown n = 39.

^2 ^Offending substances reported: insects (n = 15), drugs (n = 14), detergents (n = 9), mildew (n = 8), perfume (n = 8) and smoke (n = 5). Substances reported for less than five persons each are not specified.

The adolescents suffered to a large extent from complex allergy-like conditions, i.e. hypersensitivity to multiple offending substances and/or allergic diseases. Fifty per cent of those with hypersensitivity (n = 334/663) reported more than one kind of offending substance and 35% of those who reported allergic diseases (n = 260/739) reported more than one type of disease. Fifty-one per cent of those with allergy-like conditions (n = 471/931) reported both hypersensitivity and allergic disease.

Significantly more females than males reported allergy-like conditions (Table 2). In addition, hypersensitivity to more than one type of offending substance was more frequently reported by females than by males (55% and 43%, respectively, p < 0.01). Females reported also to a greater extent more than one kind of allergic disease (40% and 29%, respectively, p < 0.001).

Out of the 931 adolescents with allergy-like conditions, 404 (43%) reported that they had been tested for allergy by means of blood test or skin prick test (results not shown). For the majority (n = 324) of these adolescents with self-reported allergy testing, the tests had been performed during their school age years. Sixty-one per cent of those tested (n = 246/404) reported that the test results verified allergy. This figure corresponds to a 17% prevalence of self-reported verified allergy within the whole population of 1451 adolescents.

### Food hypersensitivity

In the group of 271 adolescents reporting food hypersensitivity, 139 (51%) reported allergy test results that verified some kind of allergy, albeit not necessarily food allergy (results not shown).

The most common food-induced symptoms were OAS (Oral Allergy Syndrome)-like symptoms (Table 3), i.e. itching and swelling of the lips and oral cavity, reported by 52% of the adolescents, similarly reported by females and males. Significantly more females than males reported food-induced symptoms from the skin (p < 0.001) and from the gastro-intestinal tract (p < 0.05).

**Table 3:** Food-induced symptoms, hypersensitivity to other substances besides food and allergic diseases among adolescents with food hypersensitivity

Adolescents with food hypersensitivity	Total		Females		Males		
	N = 271^1^		N = 165		N = 98		*p*-values
	N	(%)	N	(%)	N	(%)	
**Food-induced symptoms**	**271**	**(100)**	**165**	**(100)**	**98**	**(100)**	
OAS^2^-like symptoms	140	(52)	83	(50)	55	(56)	NS
skin symptoms	81	(30)	62	(38)	16	(16)	<0.001
gastro-intestinal symptoms	76	(28)	55	(33)	20	(20)	<0.05
breathing difficulties	67	(25)	38	(23)	29	(30)	NS
eye/nose symptoms	35	(13)	20	(12)	15	(15)	NS
anaphylaxis	32	(12)	21	(13)	11	(11)	NS
**Hypersensitivity to defined substances**	**208**	**(77)**	**128**	**(78)**	**72**	**(74)**	**NS**
pollen	141	(52)	79	(48)	56	(57)	NS
furred animals	125	(46)	67	(41)	53	(54)	<0.05
dust/mite	90	(33)	56	(34)	33	(34)	NS
nickel	64	(24)	54	(33)	7	(7)	<0.001
other substances^3^	26	(10)	20	(12)	6	(6)	NS
**Allergic diseases**	**210**	**(78)**	**135**	**(82)**	**70**	**(71)**	**<0.05**
asthma/wheezing	88	(33)	61	(37)	24	(25)	<0.05
eczema/rash	93	(34)	66	(40)	25	(26)	<0.05
rhino-conjunctivitis	167	(62)	103	(62)	58	(59)	NS

^1 ^Gender unknown n = 8.

^2 ^OAS = Oral Allergy Syndrome

^3 ^The substances reported for at least three persons each were: mildew (n = 4), drugs (n = 3), insects (n = 3) and perfume (n = 3).

Offending food items reported were: nuts (39%), fruit and berries (35%), peanut (32%), almond (22%), tomato (19%), carrot (16%), lactose (12%), vegetables (10%), shellfish (9%), soy (7%), milk (7%), fish (5%) and egg (5%). Substances reported for less than five per cent of the 271 adolescents are not specified. For two food items there were significant gender differences. The offending food items fruit and berries were more commonly reported by females (44% and 24% respectively, p < 0.001) and peanut was more commonly reported by males (43% and 27% respectively, p < 0.01).

A total of 63 adolescents reported food as the only offending substance. However, the majority of the 271 adolescents who reported food hypersensitivity suffered from complex allergy-like conditions that included additional offending substances besides food (77%) as well as allergic diseases (78%). In this group of food hypersensitive adolescents there were significantly more males than females who reported hypersensitivity to furred animals and as regards hypersensitivity to nickel the result was reverse (Table 3). Allergic diseases such as asthma/wheezing and eczema/rash were also significantly more often reported by the females (Table 3).

### Health-related quality of life

Adolescents with allergy-like conditions scored significantly lower on seven of the eight SF-36 health scales compared with adolescents without such conditions (Table 4). The adolescents with allergy-like conditions scored similar on the health scales whether they had reported verified allergy or not (results not shown). Moreover, Table 4 also demonstrates gender differences among the adolescents with allergy-like conditions. Females reported significantly lower SF-36 scores in seven of the eight health scales.

**Figure 1:**
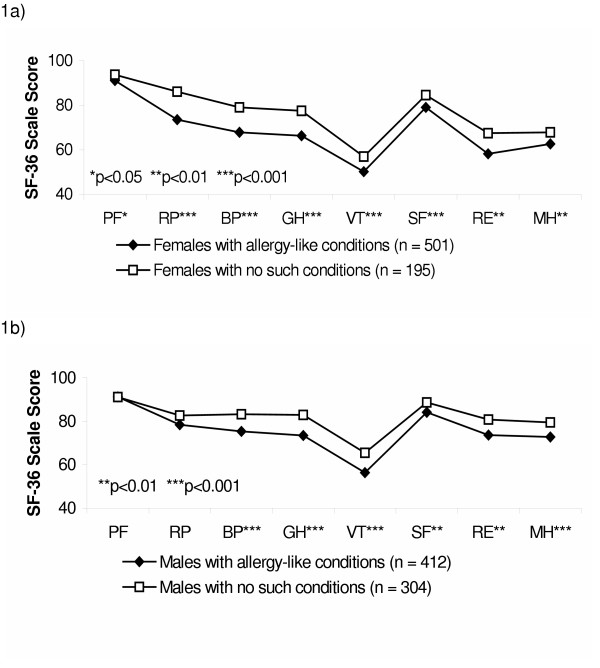
Comparison of SF-36 scores between: a) females with allergy-like conditions and females with no such conditions, and b) males with allergy-like conditions and males with no such conditions. (*Physical dimension: *PF, physical functioning; RP, role functioning-physical; BP, bodily pain; GH, general health. *Mental dimension: *VT, vitality; SF, social functioning; SE, role functioning-emotional; MH, mental health.)

When comparing females with and females without allergy-like conditions, the former group scored significantly lower on all eight scales (Figure 1a). For the males, statistically significant differences were seen for all health scales, except for physical functioning (PF) and role functioning-physical (RP) (Figure 1b).

**Figure 2:**
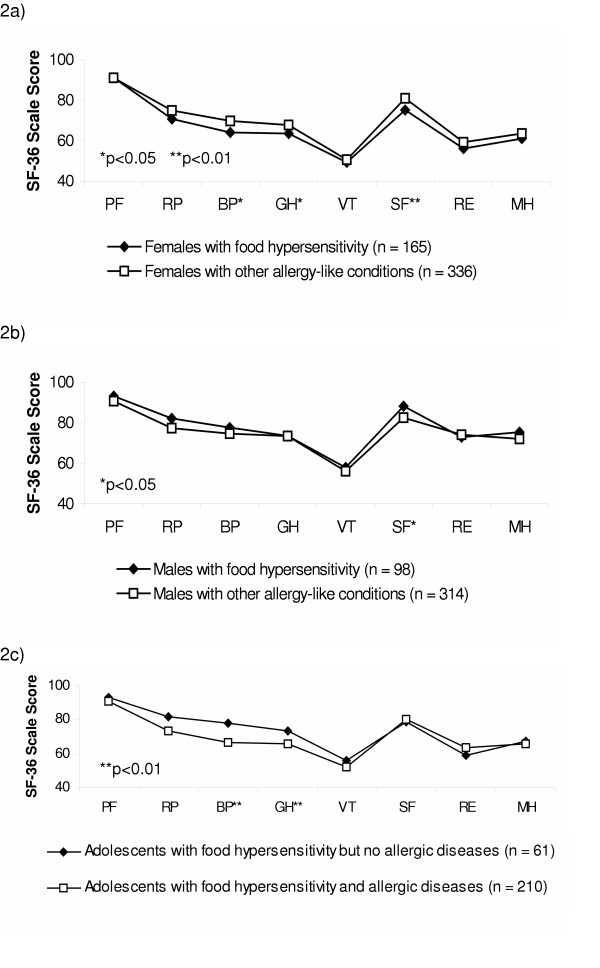
Comparison of SF-36 scores between: a) females with food hypersensitivity and females with other allergy-like conditions, b) males with food hypersensitivity and males with other allergy-like conditions, and c) adolescents with food hypersensitivity with or without allergic diseases. (*Physical dimension: *PF, physical functioning; RP, role functioning-physical; BP, bodily pain; GH, general health. *Mental dimension: *VT, vitality; SF, social functioning; SE, role functioning-emotional; MH, mental health.)

Figure 2 shows comparisons between food hypersensitive adolescents, females and males, with or without other allergy-like conditions. Females with food hypersensitivity scored significantly lower on three health scales (BP, GH and SF) compared with females with other allergy-like conditions (Figure 2a). A corresponding comparison for the males showed no HRQL-deterioration for the food hypersensitive males (Figure 2b).

When comparing food hypersensitive adolescents who reported allergic diseases (i.e. asthma/wheezing, eczema/rash and rhino-conjunctivitis) (n = 210/271) to those food hypersensitive adolescents who did not report such conditions (n = 61/271), the groups showed a similar pattern as regards the mental dimension scales of the SF-36, i.e. no statistically significant differences were found. As regards the physical dimension scales, the food hypersensitive adolescents who also reported allergic diseases scored significantly lower on BP (bodily pain) and GH (general health) (Figure 2c).

**Figure 3:**
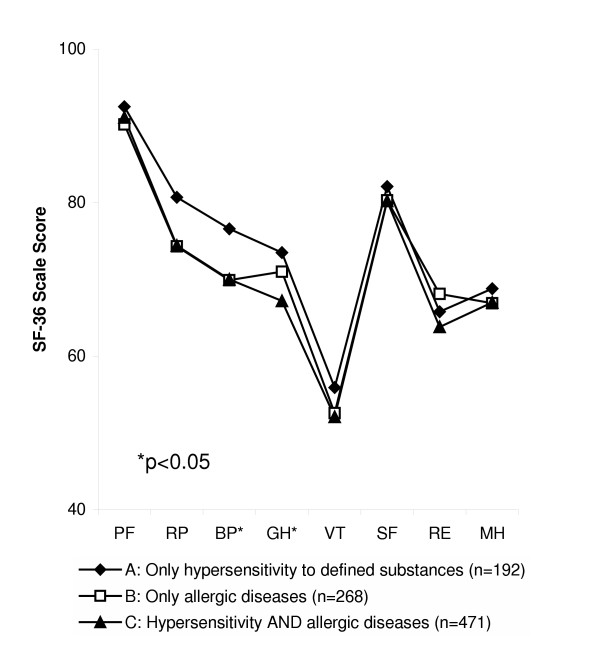
Comparison of SF-36 scores between adolescents with (A) only hypersensitivity to defined substances, (B) only allergic diseases and (C) both (p < 0.05: BP A > B and C; GH A > C). (*Physical dimension: *PF, physical functioning; RP, role functioning-physical; BP, bodily pain; GH, general health. *Mental dimension: *VT, vitality; SF, social functioning; SE, role functioning-emotional; MH, mental health.)

A comparison within the whole group of adolescents with allergy-like conditions (n = 931), i.e. between those who reported only hypersensitivity to any defined environmental substances (A), those who reported only allergic diseases (B), and those who reported both (C), showed that the three groups scored similar on the four health scales representing the mental dimension of the SF-36. As regards the physical dimension, adolescents with allergic diseases only (B) or in combination with hypersensitivity (C) scored lower on the health scales for bodily pain (BP) and general health (GH) compared with those with only hypersensitivity to defined substances (A) (Figure 3).

## Discussion

The present study focuses on self-reported allergy-like conditions among adolescents, in particular food hypersensitivity. The results show that as many as 64% of the adolescents reported allergy-like conditions, of which nearly one third reported food hypersensitivity. In most cases the allergy-like conditions were complex, i.e. included hypersensitivity to multiple offending substances and/or allergic diseases.

Compared with the prevalence for Swedish children 13–14 years of age, shown in an ISAAC-study [4], the adolescents (13–21 years) in the present study reported about the same prevalence of asthma/wheezing (15% and 16%, respectively) and of eczema (17% and 20%, respectively), but a higher prevalence of rhino-conjunctivitis (26% and 38%, respectively). The differences in prevalence of rhino-conjunctivitis between these studies might to some extent be explained by the fact that different age groups were sampled. Furthermore, the referred ISAAC-study was performed at least six years earlier than the present one. During this time span the allergy problem has been a growing concern among both children and adults and the rates may have risen [1,2].

The adolescents with allergy-like conditions generally showed significantly lower HRQL than adolescents without such conditions. Previous studies have shown that doctor-diagnosed asthma, eczema, and rhinitis have negative impacts on HRQL [10-18]. In the present study we have shown that those who reported that they suffered from these allergic diseases scored low on SF-36, regardless whether the diseases were verified by medical expertise or not. Furthermore, also the adolescents who reported hypersensitivity without having such diseases, scored low on SF-36, especially on the scales concerning mental health and emotional-social functioning. This is in accordance with previous findings in children with doctor-diagnosed peanut allergy [20,21]. Living with constant vigilance, uncertainties and risks of adverse reactions, is likely to influence HRQL in adolescents in a negative way.

The question of co-morbidity has been discussed [29], as it is not always possible to grasp what component(s) of a complex allergy-like condition affects the HRQL. Furthermore, one has to consider the possibility that the HRQL-deteriorations in these adolescents may not be a direct effect of their allergy-like conditions, but related to an overall poorer general state of health. The presence of poor social, mental or somatic health may increase the perception of allergy-like conditions. Still, irrespective of what the underlying causes are, it is evident that adolescents who experience allergy-like conditions also experience HRQL-deterioration.

In most of the comparisons between different subgroups of adolescents with and without allergy-like conditions, the SF-36 health scale for physical functioning (PF) showed no significant difference. This is noteworthy, as the physical parameter often is in focus when health-care professionals assess an individual's state of health. However, it has been previously shown that in patients with asthma/wheezing, the link between lung function and HRQL is weak [11,12] and the results of the present study indicate that the link between physical parameters and the HRQL may be weak also in other kinds of allergy-like conditions. Hypersensitivity may perhaps be considered as a practical, emotional and psychosocial health problem – not primarily a physical. It has been shown, however, that HRQL-deterioration among peanut allergic children is related to anxiety and fear for adverse reactions [20,21]. Moreover, the adolescents with a non-severe chronic allergic disease may be well adapted to the disease, physiologically and/or psychologically, so that the disease as such has no significant impact on their physical quality of life.

In the present study we show that in adolescence, significantly more females than males experienced not just asthma/wheezing but also eczema/rash, rhino-conjunctivitis, and hypersensitivity to food, dust/mite and nickel. The females presented more complex allergy-like conditions compared with the males. In addition, females with allergy-like conditions showed more severe HRQL-deterioration compared with the males. It is known that female gender among adults implies a larger report of burden of health problems in general [30] and SF-36 Swedish normative data show that females 15–19 years of age score lower compared with males in this age group [27]. Thus, the gender differences with respect to allergy-like conditions were in accordance with a known pattern within the health-and-gender-field. Biological, hormonal and socio-cultural explanations to gender differences in asthma and allergy have been discussed [24,31,32] as well as possible gender biased diagnostic practices [33-35] such as underdiagnosis of females due to gender differences in disease severity. Further research in this area is needed so that health-care professionals, school personnel, relatives and friends can improve their care and support given to both females and males suffering from allergy-like conditions.

Seventeen per cent of the adolescents, of whom the most part were tested during school age, reported positive allergy test results. It can be assumed that there were some additional adolescents with doctor-diagnosed allergy but without verifying test results. Thus, the total of adolescents doctor-diagnosed as having allergy may well be more than 17%. However, in the present study the focus was on self-reported (perceived or verified) allergy-like conditions. The mean SF-36 scores did not differ whether the adolescents reported objectively verified allergy or not. The lack of difference in HRQL was not surprising as a diagnostic test that verifies allergy says nothing about the individuals experience or the severity of the allergic condition.

### Food hypersensitivity

Most food allergies are something that children outgrow [36] and adverse reactions to food should consequently be a problem that decreases with age. The prevalence of food allergy is estimated to 5–8% in children and 1–2% in adults [37]. However, figures of perceived reactions to food may well be over 20% [6]. In the present study, 19% of the adolescents did report adverse reactions to food. This high figure may be explained by the fact that the reactions were self-perceived and not necessarily doctor-diagnosed and that the figure includes all kinds of food hypersensitivity regardless of the mechanisms behind the adverse reactions. The existing diagnostic methods for food hypersensitivity are not sufficient and the underlying mechanisms of perceived food hypersensitivity are not always known [38]. Nevertheless, it is noteworthy that almost every fifth adolescent perceive herself or himself as food hypersensitive and subsequently avoid certain food items.

Food items constituted a considerable part of the offending substances reported in this study and up to 41% of all adolescents with hypersensitivity specified at least one food item as an offending substance. Professional counselling and diagnostic procedures may to some extent be able to help the adolescents to reduce their food avoidance. Yet, this kind of perceived allergy-like condition – regardless of what the underlying mechanisms were – was evidently associated with HRQL-deterioration and the adolescents' experiences deserve sincere attention. Previous research show that food allergy in a child implies disruption in daily life and HRQL-deterioration for both the child and the family [19-21].

A pattern emerged in this group of food hypersensitive adolescents, showing a great deal of hypersensitivity to pollen, rhino-conjunctivitis and OAS-like symptoms, which is in accordance with the well known cross-reactivity of pollen and food allergens [39]. Significantly more males than females reported additional hypersensitivity to furred animals. To our knowledge this has not been reported before but may be associated with the higher atopy prevalence among males [22]. The results also pointed to a possible gender difference in what offending food items females and males, respectively, experience. Further research is needed to elucidate such a phenomenon.

More than half of the adolescents with food hypersensitivity reported positive allergy tests, but as a consequence of how the questions in the survey were asked, it is not known if they were verified as allergic specifically to food. However, a vast majority of the food hypersensitive adolescents did undoubtedly suffer from complex allergy or allergy-like conditions, which included multiple types of hypersensitivity and/or allergic diseases. This should require competence in health-care when trying to tackle the adolescents' multifaceted problems, including food hypersensitivity.

### Limitations

The extensive number of adolescents who participated in the present survey, together with a very high answer rate, makes the results reliable. However, the sample of adolescents used in this study may limit the generalizability as the socio-demographic distribution in this particular municipality was slightly above in socio-economics and slightly below in number of immigrants compared with the country as a whole. Additional studies are warranted to confirm the results.

The results of the present study emanate exclusively from adolescents' statements. There is always a risk that the respondents involved do not correctly remember things that were asked for in a questionnaire. However, our main interest was in adolescents' experiences of allergy-like conditions during the past twelve months and daily functioning during the past four weeks. Remembering correctly over a longer period of time was only of importance in questions about allergy testing. It seems likely that the adolescents would remember such events as skin prick tests or blood tests during their school age, although tests carried out in early childhood might have been unknown or forgotten.

The magnitude of allergic diseases was measured by means of ISAAC-questions. The ISAAC questionnaire, which asks for events during the past 12 months, has been used in many countries all over the world and for many years. Its results constitute basis for international comparisons and it can be considered well validated. However, hypersensitivity items are not included in the ISAAC questionnaire. The questions in the present study about hypersensitivity were developed specifically for the present study and a pilot test was performed. Only lexical adjustments were needed.

It could be discussed if the fact that the questionnaires asked for events during two distinct periods: 12 months (allergy-like conditions/ISAAC-questions) and 4 weeks (daily functioning/SF-36) might have biased the present results. It is also possible that an allergy-specific HRQL measure instrument would give another picture of the physical scale in relation to allergy-like conditions.

## Conclusions

The prevalence of self-reported allergy-like conditions among adolescents was high – 64%. Significantly more females than males reported allergy-like conditions and females with allergy-like conditions showed more severe HRQL-deterioration compared with males with such conditions. The results indicate a need to consider not merely physical consequences but also the psychosocial quality of life impact of allergy-like conditions among both females and males. Further research is needed to elucidate the reasons behind the gender differences in this area.

Most adolescents suffered from complex allergy-like conditions that included multiple types of hypersensitivity and/or allergic diseases. Food items constituted a considerable part of the offending substances reported. When attending to a young individual who suffers from an allergy-like condition, the whole syndrome should be in focus – not only one specific offending substance, or one specific hypersensitivity or allergic disease. A team approach accompanied by an understanding of how allergy-like conditions impair the quality of life may improve the management of the adolescent's health problems, both in health-care services and in schools.

## Authors' contributions

BM and GN conceived of the study. All authors made substantial contributions to conception, planning and design. BM carried out the acquisition, analysis and interpretation of data. BM drafted the manuscript. GN and SA have been involved in revising it critically for important intellectual content. All authors read and approved the final manuscript.
